# Production, characterization, and epitope mapping of monoclonal antibodies of ribosomal protein S3 (rpS3)

**DOI:** 10.1080/19768354.2021.1980100

**Published:** 2021-10-06

**Authors:** Woo-Sung Ahn, Tae-Sung Kim, Yong Jun Park, Young Kwang Park, Hag Dong Kim, Joon Kim

**Affiliations:** a Laboratory of Biochemistry, Division of Life Sciences, Korea University, Seoul, Republic of Korea; b HAEL Lab, Korea University, Seoul, Republic of Korea

**Keywords:** Ribosomal protein subunit small 3, epitope mapping, mAb, peptide synthesis, direct ELISA, sandwich ELISA, linear form epitope, conformational epitope, immunoprecipitation

## Abstract

Ribosomal protein S3 (rpS3), a member of 40S small ribosomal subunit, is a multifunctional protein with various extra-ribosomal functions including DNA repair endonuclease activity and is secreted from cancer cells. Therefore, antibodies with high specificity against rpS3 protein could be useful cancer biomarkers. In this study, polyclonal antibody (pAb) and monoclonal antibodies (mAbs) were raised against rpS3 protein and epitope mapping was performed for each antibody; the amino acid residues of rpS3 were scanned from amino acid 185 to 243 through peptide scanning to reveal the epitopes of each mAb. Results showed that pAb R2 has an epitope from amino acid 203 to 230, mAb M7 has an epitope from amino acid 213 to 221, and mAb M8 has an epitope from amino acid 197 to 219. Taken together, novel mAbs and pAb against rpS3 were raised and mapped against rpS3 with different specific epitopes.

## Introduction

Monoclonal antibodies (mAbs) are useful biological tools for various analytical applications, including clinical chemistry, food analysis, and environmental monitoring. In addition, antibodies are increasingly used as human therapeutics. Immunization of animals, mainly mice, in combination with hybridoma technology, is still the most common method for generating mAbs. Irrespective of the intended application, selection of high-affinity mAbs were often preferred, and an efficient hybridoma screening procedure is a critical step that usually must be completed in a short time (Burrin and Newman 1991; Jang et al. 2020). Therefore, an ideal screening method should be fast, reliable, and easy to perform, especially if the laboratory does not have an equipment for performing automated immunoassays. The screening method should clearly detect high-affinity mAbs with a minimum of both false positives and false negatives. In addition, useful screening results must be obtained relatively independent of the mAb concentration in the supernatants because optimization of the ELISA parameters (such as supernatant dilution and coating conjugate dilution) prior to the screening is usually time-consuming or even impossible, especially as the screening often involves a single measurement per mAb. This study relates to a mAb of human rpS3 (ribosomal protein small subunit 3, https://www.ncbi.nlm.nih.gov/gene/6188), particularly, a mAb produced by using recombinant protein expressed and purified with *E. coli* using pET-15b vector.

For cells to survive, proteins must be continuously synthesized. Ribosomes, the protein synthesis machinery of all living cells, are composed of several rRNAs and ribosomal proteins. Ribosomal protein S3 (rpS3), also known as ribosomal protein uS3, is a member of 40S small ribosomal subunit encoded by the *RPS3* gene. RpS3 has a mass of 26.69 kDa and an amino acid length of 243. RpS3 is located on the outer surface of the 40S subunit of the ribosome and plays an important role in protein synthesis *in vivo* (Westermann et al. 1979). The human *RPS3* gene is located on 11q13.3-q13.5 on human chromosome 11. Also, rpS3 is reported to be involved in the regulation of its own mRNA transcription (Polakiewicz et al. 1995; Lee et al. 2006; Lim et al. 2002; Kim et al. 2010). The rpS3 protein has extra-ribosomal functions; it is first discovered to be UV endonuclease III, which functions as an endonuclease to repair DNA damage caused by ultraviolet light (UV) (Kim and Linn 1989; Kim et al. 1995). Furthermore, it was recently discovered that the rpS3 protein associates with the TFIIH complex and positively regulates UV-damaged nucleotide excision repair by assisting XPD helicase and increasing the turnover rate of TFIIH complex (Park et al. 2021). Also, rpS3 appear to aid damaged DNA processing by cleaving apurinic/apyrimidic (AP) sites; that is, when DNA is damaged, the base of the damaged area is cut off with the function of another DNA glycosylase to form an AP site, and then the AP site is cleaved by an AP endonuclease activity of rpS3. The AP endonuclease activity of rpS3 functions as a lyase through β-elimination. In fact, rpS3 appears to have a wide range base-damage-endonuclease activity which recognizes various kinds of damaged lesions, including AP sites, thymine glycols and pyrimidine dimers (Kim et al. 2005; Yang et al. 2019; Park et al. 2020). Therefore, rpS3 can cleave the phosphodiester bonds at the pyrimidine dimer generated by UV irradiation. In addition, rpS3 is overexpressed in colorectal cancer cells, which suggests that there may be a relationship between the occurrence of colorectal cancers and rpS3 (Pogue-Geile et al. 1991).

Epitope mapping is the process of experimentally identifying the binding site, or ‘epitope,’ of an antibody on its target antigen (usually a protein) (Dejana and Corada 1999; Westwood and Hay 2001; Davidson and Doranz 2014). The identification and characterization of antibody binding sites aid in the discovery and development of new therapeutics, vaccines, and diagnostics (Gershoni et al. 2007; Dutton 2016; Saphire et al. 2018). Epitope characterization can also help elucidate the mechanism of antibody binding (Davidson et al. 2015) and can enforce intellectual property (patent) protection for the novel antibodies (Deng et al. 2018; Ledford 2018). Experimental epitope mapping data can be incorporated into robust algorithms to facilitate the *in silico* prediction of B-cell epitopes based on sequence and/or structural data (Schreterova et al. 2017; Leclair and Lim 2020). Epitopes are generally divided into two classes: linear and conformational. Linear epitopes are formed by a continuous sequence of amino acids in a protein. Conformational epitopes are composed of amino acids discontinuous in the protein sequence but brought together upon three-dimensional protein folding. B-cell epitope mapping studies suggest that most interactions between antigens and antibodies, particularly autoantibodies and protective antibodies (e.g. in vaccines), rely on binding to conformational epitopes (Dejana and Corada 1999; DeLisser 1999; Potocnakova et al. 2016).

MAbs serve as useful diagnostic tools and can serve as probes for cellular and macromolecular investigations. (Nelson et al. 2000). In this study, we purified mAb raised against rpS3 peptides from amino acid residues 185 to 243 to identify specific epitope binding sites. We also characterized the production and epitope mapping of single clones using *in vitro* assays such as immunoblot assay, ELISA, and immunoprecipitation. Hybrid technology has the potential to provide an infinite supply of high-quality monospecific mAbs (Shakil et al. 2001; Banik et al. 2017).

## Materials and methods

### Overexpression and purification of rpS3 recombinant proteins

Human rpS3 wild-type and mutants were generated using polymerase chain reaction (PCR) and subcloned into the vector pGEX-5T-1 (Pharmacia, Sweden), using *EcoRI* and *XhoI* restriction enzyme sites introduced into sense and antisense primers, respectively. Information regarding all primers used is provided in Table S1a and S1b. *E.coli* strain BL21 transformed with glutathione S-transferase (GST)-rpS3 or mutant plasmids was cultured overnight at 37°C as a starter, added to fresh media, and cultured until OD_600_ = 0.6. Then, a final concentration of 0.2 mm of isopropyl β-D-1-thiogalactopyranoside (IPTG) was added to the culture and incubated at 30°C for 4 h. Cells were harvested and resuspended in PBS (pH 7.2, Hyclone, GE Healthcare Inc. CA, USA) containing protease inhibitors (5 μg·mL^−1^ leupeptin, 10 μg·mL^−1^ aprotinin, and 1 μg·mL^−1^ PMSF). Cells were sonicated and centrifuged at 15,000*g* for 30 min at 4°C. Glutathione-agarose resin (Thermo Scientific, Waltham, MA, USA) was added to the supernatant and incubated at 4°C for 4 h. Resins were washed three times with PBS, and the proteins were eluted with 10 mM of reduced L-glutathione (Sigma-Aldrich, St. Louis, MO, USA) and 50 mm Tris-Cl, pH 8.0. His-rpS3 was subcloned into pET-21a, transformed into BL21 cells, and cultured as described above. Cells were harvested and resuspended in lysis buffer (5 mM imidazole, 100 mM NaCl, 0.1 mM EDTA, 50 mM Tris-Cl, pH 8.0) containing protease inhibitors. Centrifugation was performed at 12,000*g* at 4°C for 20 min to obtain a supernatant, which was bound at 4°C for overnight with GST-beads. The beads were centrifuged at 900*g* at 4°C for 5 min to remove the supernatant and washed five times with 1 mL of PBS. After adding 200 µL of PBS, the GST-beads were allowed to stand on ice for 1 h. Of the 200 µL thus obtained, 10 µL was quantified with nanodrops. The fragments of recombinant rpS3 were identified using SDS-PAGE.

### Production of monoclonal antibodies

The mAb M8 was produced and purified by conventional hybridoma production methods, as previously described (Malito et al. 2013; Malito et al. 2014). The mAb was purified from culture supernatants by Protein G affinity columns (GE Healthcare). Purified mAbs were further confirmed by ELISA, Western blotting, as described below.

### Peptide synthesis

The sequence of the human rpS3 peptide and its fragments were synthesized for epitope mapping by Lugen Sci Co., Ltd., Seoul, Korea. In this study, 52 rpS3 fragments for epitope mapping were synthesized. Each fragment was composed of 15 amino acids (Table S2). The fragments formed a series of sequences, each member of the series being shifted along the rpS3 sequence by an interval of 1 amino acid residues from the previous member. The stock solution was dissolved in autoclaved distilled water at a concentration of 1 mg/mL, and stored at −70°C.

### Western blotting

Culture media and cell lysates were separated on a 12% polyacrylamide gel, transferred to a 0.45 μm PVDF membrane (Merck Millipore, Germany) using a semi-dry blotting protocol, and blocked for 1 h at room temperature (about 22°C) with 5% BSA in Tris-buffered saline with 0.1% Tween® 20 detergent (TBST). The primary antibody was diluted 1:2000 and incubated at 4°C for overnight. After washing three times with TBST, the secondary antibody, anti-mouse Horseradish peroxidase (HRP), was diluted 1:2000 in TBST, incubated at room temperature, and washed three times with TBST. Cells were incubated with ECL Western blotting Reagent (GE Healthcare, UK), as suggested by the manufacturer. The signal was detected on a Super HR-HA 30 film (Fuji, Japan).

Mouse anti-rpS3 monoclonal antibody, M7, and rabbit anti-rpS3 polyclonal antibody, R2 were purified and stored in the laboratory. Commercial mouse anti-rpS3 monoclonal antibody (RPS3) was purchased from MyBioSource, Inc. (San Diego, CA, USA). HRP-conjugated goat antirabbit and goat anti-mouse secondary antibodies were purchased from

Jackson ImmunoResearch Inc. (West Grove, PA, USA). A mouse anti-GST antibody (Santa Cruz Biotechnology, CA, USA) was used to detect a GST-tagged rpS3 recombinant protein fragments.

### Cell culture and siRNA transfection

HT1080 fibrosarcoma cell lines were seeded into 6-well plates at a density of 1 × 10^5^ cells. After 24 h, at approximately 70% confluency, the cells were transfected. For Lipofectamine-mediated transfection, 10 μL of 20 μM siRNA777 was mixed with 4 μL of Lipofectamine RNAi Max (Invitrogen, CA, USA) in Opti-MEM (Gibco, CA, USA). Lipofectamine RNAi Max was transfected in parallel plates with the same concentration of scrambled siRNA777 as a control. After 24 h, cells were extracted for gene expression analysis. Before loading on SDS-PAGE, cleared lysates were mixed with Laemmeli buffer and boiled at 95°C for 5 min.

### Direct ELISA assay

Polystyrene 96-well microtiter plates (Costar, NY, USA) were coated with a solution of rpS3 recombinant protein (10 µg/mL) in PBS and the plate was incubated at 4°C for overnight. The plate was washed four times with PBST (0.01% Tween-20 in PBS) and blocked with 4% bovine serum albumin (BSA, GenDEPOT, Korea) containing in PBST at 37°C for 2 h (400 µL/well). mAb M7, pAb R2, and supernatant of hybridoma clones (also named M8) were added to each well (100 µL/well) and incubated at 37°C for 2 h. After washing the plate four 4 times with PBST, 100 µL of HRP-conjugated anti-mouse antibody (1:2000) was added and the plates were incubated at 37°C for 2 h. After washing three times, 3,3′,5,5′-tetramethylbenzidine (TMB) liquid substrate (100 µL/well, Thermo Scientific, USA) was added and the reaction was allowed to proceed for 5 min. Enzymatic reactions were stopped with a stop solution (0.16 M sulfuric acid, Thermo Scientific, USA) (100 µL/well). The absorbance of each well at 450 nm was measured using an automated ELISA reader (3550 Microplate reader, Biorad, Richmond, CA, USA).

### Direct ELISA of synthesized peptides

Polystyrene 96-well microtiter plates were coated with a solution of synthesized peptides (10 µg/mL) in PBS and the plate was incubated overnight at 4°C. After washing the plate four times, the plate was blocked with 4% BSA containing in PBST at 37°C for 2 h (400 µL/well). mAb M7, pAb R2 and hybridoma clones supernatant were added to each well (100 μL/well) and incubated at 37°C for 2 h. After washing the plate four times with PBST, 100 µL/well of HRP-conjugated anti-mouse antibody (1:2000) was added and the plates were incubated at 37°C for 2 h. After washing three times, TMB liquid substrate (100 µL/well) was added, and the reaction lasted for 5 min. The reactions were stopped with a stop solution (100 µL/well). The absorbance of each well at 450 nm was measured using an automated ELISA reader. Hybridoma clones with high absorbance were selected for antibody purification.

### Sandwich ELISA

Capture Ab was diluted to 2 µg/mL in PBST. Then, the solution (100 µL/well) was coated onto the wells of the ELISA plate. Wells were washed four times with PBST. PBST containing 4% BSA was added at 400 µL/well and incubated at 37°C for 1 h 30 min. The wells were washed four times with PBST. The primary antibody was diluted to 1:5000 in PBST containing 1% BSA, and 100 µL/well was added. The plate was incubated at 37°C for 2 h. At the end of incubation, plates were washed four times with PBST. One hundred microliter of the peptide or recombinant protein diluted at a concentration of 100 ng/mL was added to each well of the plate, and the plate was maintained at 37°C for 1 h. Finally, the plates were washed three times with PBST. HRP-conjugated rIgG or mIgG was diluted to 1:2000 in PBST containing 1% BSA, and 100 µL was added to each well and incubated at 37°C for 1 h. After 1 h, cells were washed three times with PBST. The TMB substrate (100 µL/well) was added for color development. Absorbance was measured at 450 nm using an ELISA plate reader.

### Competitive ELISA

This assay followed the same protocol as described for the direct ELISA, rpS3 recombinant protein fragments were coated at a concentration of 10 μg/mL (100 μL/well) and incubated at 4°C for overnight. The next day, 4% BSA in 0.1% PBST (400 μL/well) was blocked at 37 ℃ for 1 h. And, it was washed with 0.1% PBST (400 μL/well) for four times and blocked with 4% BSA in PBST (400 μL/well) at 37°C for 1 h 30 min. Then, washed with 0.1% PBST (400 μL/well) for four times. And, pAbs R2, rIgG (0.5 μg/mL, 100 μL/well) as primary antibodies were incubated at 37°C for 1 h 30 min. Thereafter, 0.1% PBST (400 μL/well) was washed four times. Secondary antibody, HRP-conjugated rIgG (100 μL/well) was diluted to 1:5000 in PBST containing 1% BSA and incubated at 37°C for 1 h. After, wells were washed three times with PBST. The TMB substrate (100 µL/well) was added for color development. Absorbance was measured at 450 nm using an ELISA plate reader. GST-S3-P was used as a positive control.

### Immunoprecipitation

Cells were collected in PBS, centrifuged, lysed in SDS-sample buffer without dye (see Western blotting above) and diluted 10 times with PBS containing 0.5% NP40 (Desterro et al. 1998) with protease inhibitors. Cell lysates were incubated for 15 min on ice and centrifuged at 12,000*g* for 10 min at 4°C. Supernatants were incubated with 5 μg/mL of rpS3 specific monoclonal antibodies or mouse IgG2b for overnight at 4°C. Protein agarose A (GE Healthcare) was added, incubated for 3 h and washed three times with ice-cold lysis buffer containing 0.5% NP40 and protease inhibitors. Proteins were eluted from protein agarose A beads by boiling in SDS sample buffer. All steps were carried out at 4°C with rocking unless stated otherwise. Immunoprecipitated proteins were analyzed using SDS-PAGE and Western blotting, as described above. Anti-Mouse IgG2b antibody was purchased from Abcam (Cambridge, UK).

## Results

### Each monoclonal antibodies to rpS3 recognize specific epitopes

**Figure 1.**
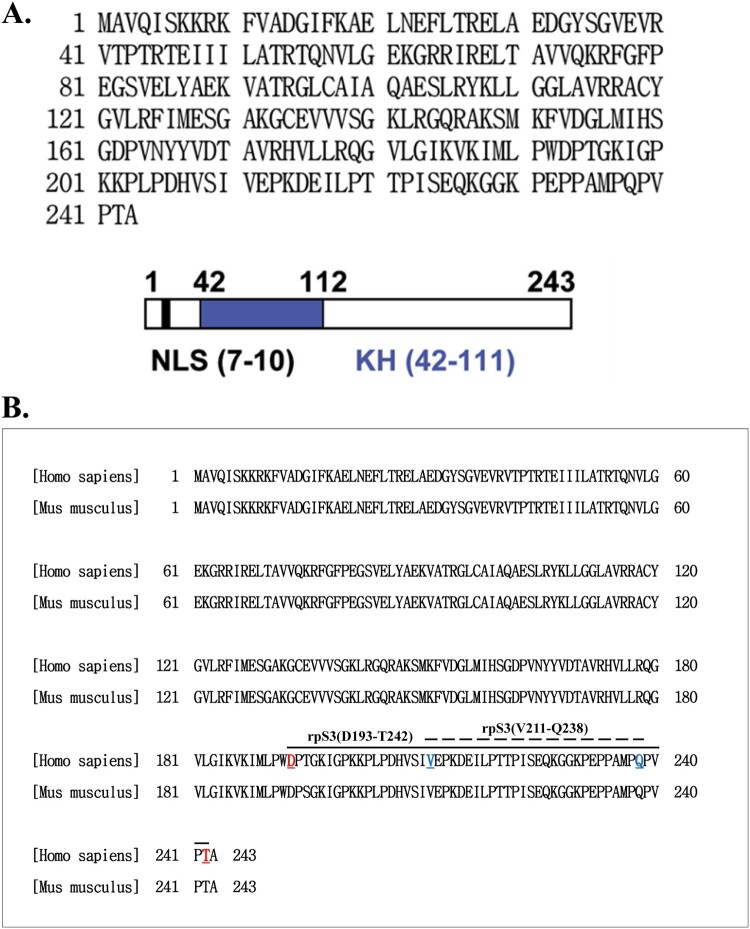
The amino acid sequence and a diagram of rpS3. (A) Amino acid sequence of human 40S ribosomal protein S3 (upper panel). Schematic representation of human rpS3. The gray area represents the N-terminal apoptotic domain. The black box indicates the C-terminal amino acids, which are sufficient for the repair endonuclease activity of rpS3. There are domains, represented by blank boxes, in both termini that appear to regulate apoptotic activities in a negative manner. KH domain is a highly conserved motif found in RNA binding proteins (down panel). (B) Ribosomal protein S3 protein sequence alignment of *Homo sapiens* (NP_001243731.1) and *Mus musculus* (NP_036182.1).

RpS3 consists of 243 amino acids with a nuclear localization signal (NLS) domain and a KH domain (Figure 1(A)). RpS3 is a component of the 40S ribosomal subunit, but it also has extraribosomal functions, such as in DNA repair (Hegde et al. 2004). The identity of human (NP_001243731.1) and mouse (NP_036182.1) rpS3 proteins were confirmed using Blastp (https://blast.ncbi.nlm.nih.gov/). The identity of both human and mouse rpS3 proteins were 100% (Figure 1(B)). Several methods for epitope characterization rely on the functional binding of the mAb to the antigen or its derivatives. One approach is to limit the scope of epitope mapping by testing the ability of the antibody to continue binding to fragments of the antigen. Thus, binding assays, including ELISA, dot blot, or Western blot assays, can provide insights into the location of the epitope within the gross anatomy of the antigen (Gershoni et al. 2007).

**Figure 2.**
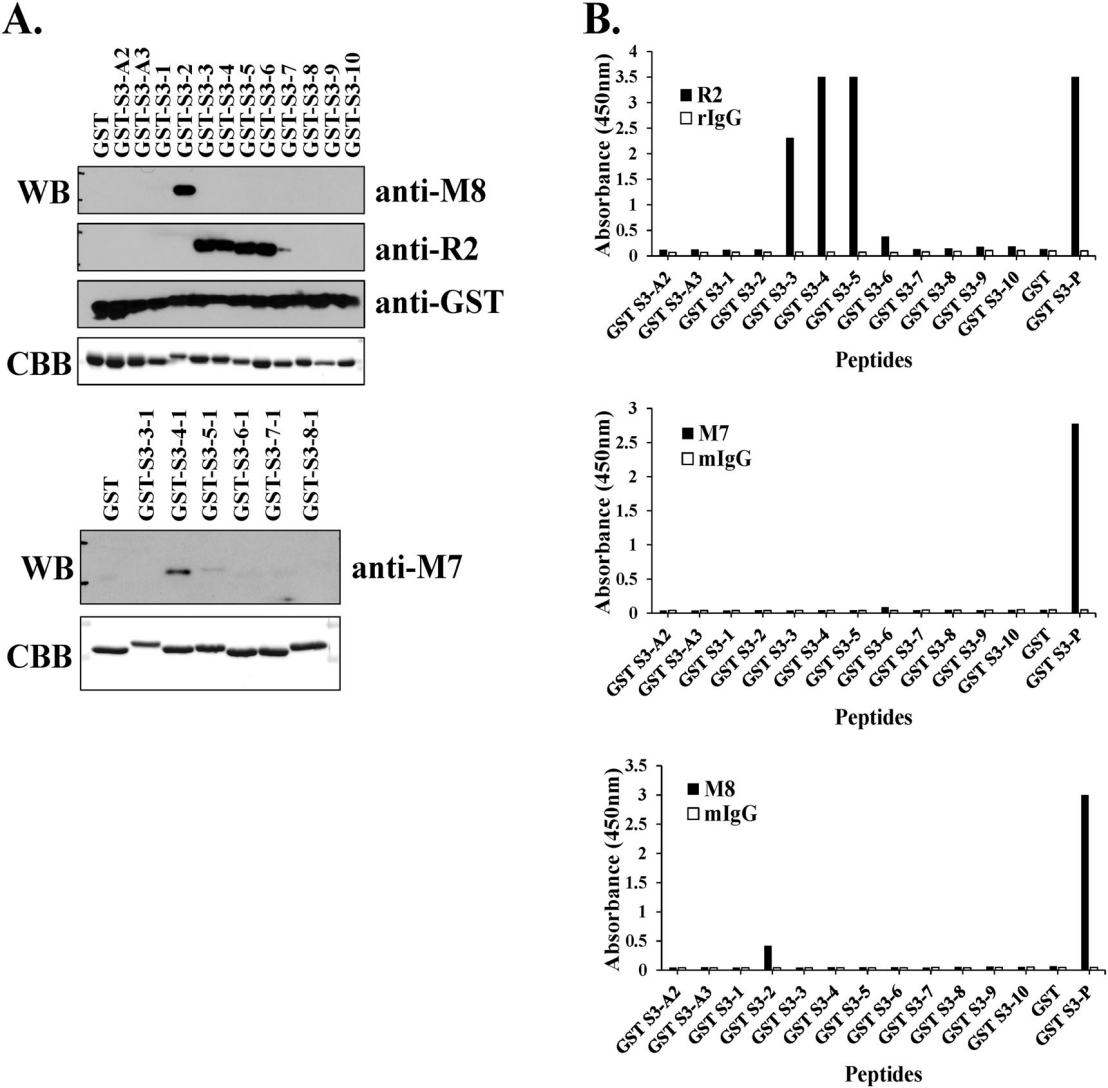
Identification of epitope binding region using monoclonal antibodies against rpS3. (A and B) Western blotting was performed using hybridoma clone supernatant M8 and specific mAb M7, pAb R2 against rpS3 using the GST-rpS3 recombinant protein purified in the previous experiment as an antigen (Table S1). After incubated with anti-M7 (1:500) at 4°C for overnight, anti-R2 (1:2000), and anti-M8 (hybridoma clone supernatant) as the first antibody, the second antibody is anti-Mouse (M7, M8; 1:2000) and anti-rabbit (R2; 1:2000) were used. Recombinant protein GST was used as a negative control. Recombinant protein GST-S3-P(C-terminal region) was used as a positive control. (C and E) Specific antigens were detected in the sample using direct ELISA assay, and the specificity of each antibody was confirmed. Twelve GST tagged overlapping recombinant proteins of rpS3 were coated onto a plate, and 100 μL of mouse monoclonal clone was added at each well. The wells were probed with horseradish peroxidase (HRP) conjugated goat anti-mouse antibody mIgG (1:2000) and subsequently developed by adding 100 μL of chromogenic substrate. The optical density was measured at 450 nm. It was confirmed that the mouse monoclonal clone targets the epitope binding site of rpS3 and is therefore specific for rpS3 as described in the Supplementary experimental (Figure S2) procedure and GST-S3-P was used as a positive control. GST (empty vector) was a negative control.

The binding affinity and recognition sites of the hybridoma clone supernatant, pAb R2 and mAb M7 for the antigen-purified GST-rpS3 recombinant proteins were confirmed by Western blotting. As shown in Figure 2, in the case of pAb R2, binding affinity from amino acids 209 to 228 of rpS3 was observed, and in particular, the supernatant of hybridoma clone M8 specifically recognized amino acids 205–212 of rpS3 (Figure 2(A), upper panel). In the case of mAb M7, the amino acids 215–222 were recognized (Figure 2(A), lower panel). In addition, the recognition sites of mAbs were identified using direct ELISA (Figure 2(B)). Direct ELISA using M8 and pAb R2 in Figure 2(B) reflected the results of Western blotting (Figure 2(A)). The hybridoma clone M8 appears to have a low affinity because it was in the state before antibody purification. However, while Western blotting in Figure 2(A) showed epitope recognition for mAb M7, the direct ELISA in Figure 2(B) showed different results. From these results, it was evident that mAb M7, pAb R2 and hybridoma clone M8 against rpS3 recognize different epitope sites. In addition, the results obtained for mAb M7 are likely to be accurate but may not be confirmed through direct ELISA.

### Pab R2 has conformational epitopes at rpS3 (215–222 aa) and (219–226 aa)

Linear epitopes were identified in the previous results. In particular, it was observed that pAb R2 was present among several epitopes (Figure 2(A,B)). To determine the site of ligand binding and competition with Ab, a competition ELISA was performed to approximate the position of Ab binding. The Ab sets can be divided into those that compete with a ligand (or a specific Ab) and those that do not. This method is called ‘competitive Ab binding’ (Ladner 2007).

We compared the inhibition of the binding of specific pAb R2 to the target antigen by binding the antibody to the competing antigen in the coated antigen, i.e. rpS3 (193-242 aa) and rpS3 (211-238 aa) recombinant protein fragments.

**Figure 3.**
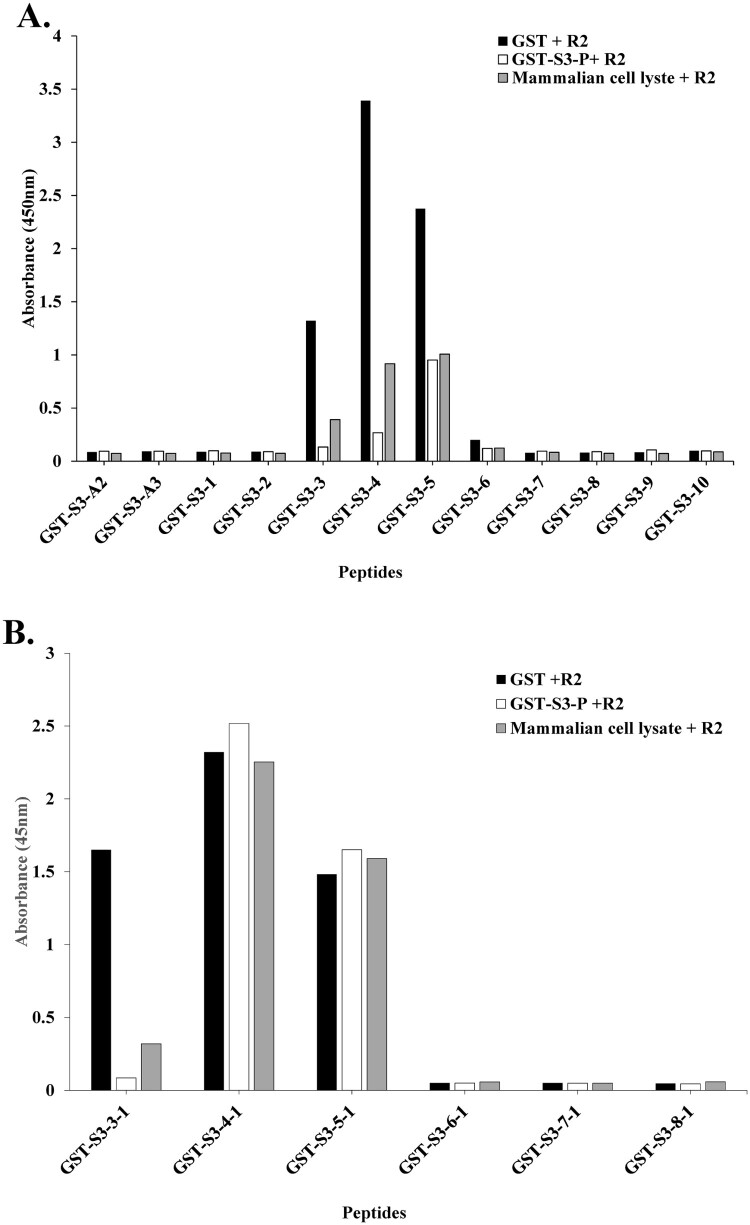
The conformational epitope of pAb R2 was identified using competitive ELISA. (A) Competitive ELISA assay was performed using 12 GST tagged overlapping recombinant protein fragments of rpS3(193-242 aa). (B) Competitive ELISA assay was performed using six GST tagged overlapping recombinant protein fragments of rpS3(211-238 aa). GST-S3-P was used as a positive control. GST (empty vector) was a negative control.

As shown in Figure 3(A), the competing antigen, GST + R2, did not bind, so it was found that the binding of the specific pAb R2 to the target antigen was not inhibited. On the other hand, in mammalian cell lysate + R2, GST-S3-3, - 4, -5 (209-216, 213-220, 217-224 aa), it was found that binding to the target antigen was inhibited at a level of about 30-50% compared to GST + R2. In particular, it shows that the binding of pAb R2 to the target antigen is inhibited by the competing antigen, GST-S3-P + R2.

In addition, as shown in Figure 3(B), uniform-binding affinities were exhibited with weak competitive Ab binding to the target antigen across GST-S3-4-1 (215-222 aa) and -5-1 (219-226 aa).

These results suggest that binding to the target antigen is inhibited by competing antigens (GST-S3-P and mammalian cell lysate). In addition, it was confirmed that the specific pAb R2 recognizes a conformational epitope (Figure 3(A,B)).

### Epitope mapping of specific monoclonal antibodies (mAbs) against rpS3 by synthesized peptides

**Figure 4.**
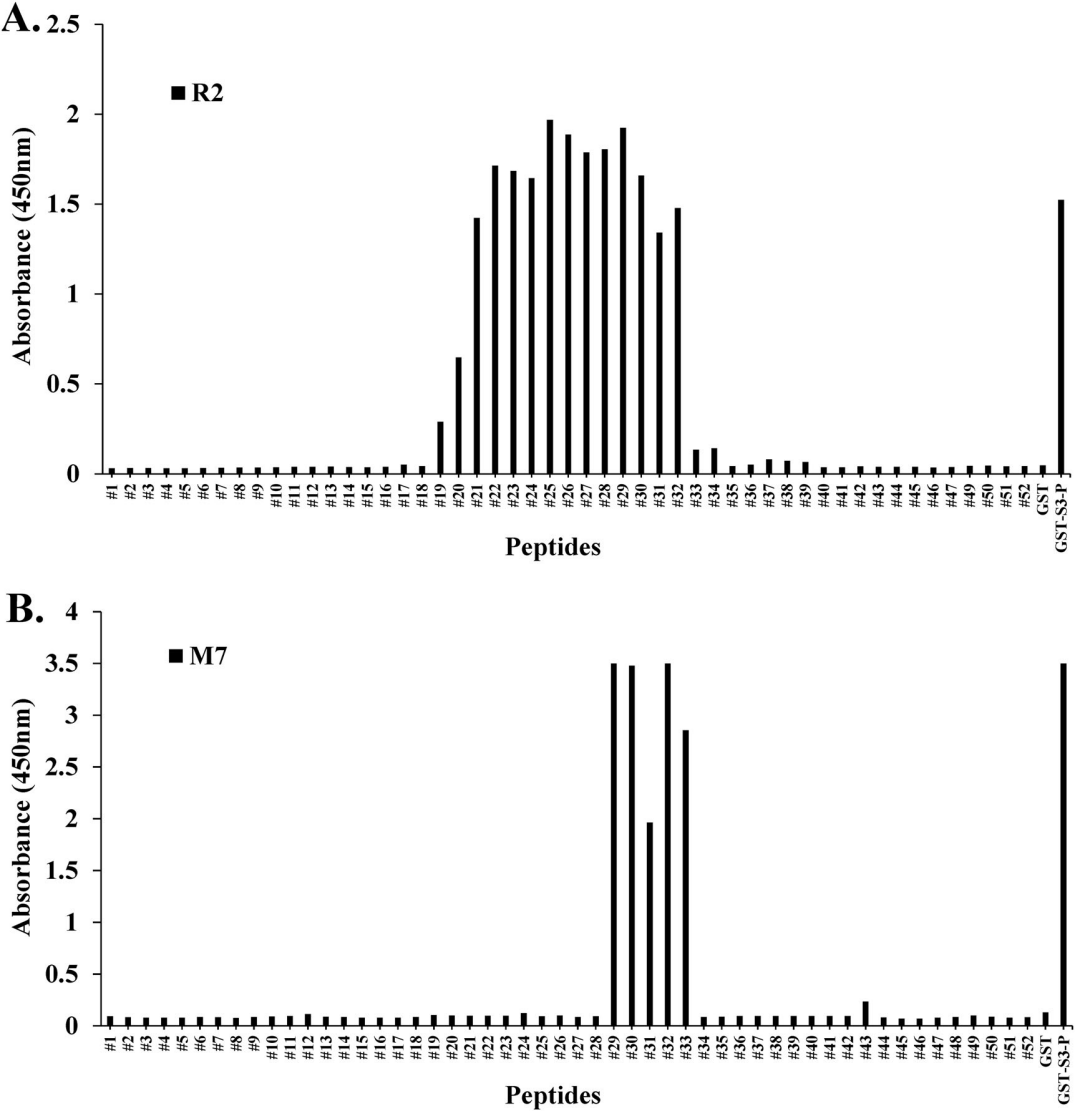
Epitope mapping of mAb M7 and pAb R2 in synthesized peptides. (A) and (B) To perform peptide scanning, 52 peptides designed with rpS3 (185-243) were synthesized with overlapping 15mer-long peptides and 14mer amino acids. Epitope mapping of (A) mAb M7 and (B) pAb R2 in the synthesized peptides was performed using direct ELISA. Synthesized peptides were coated on a 96-well plate at a concentration of 10 μg/mL and incubated at 4°C for overnight. This was blocked with 4% BSA in 0.1% PBST (400 μL/well) the next day at 37°C for 1 h. After washing four times with 0.1% PBST (400 μL/well), 4% BSA in PBST (400 μL/well) was blocked at 37°C for 1 h. In addition, washed with 0.1% PBST (400 μL/well) for four times. And pAb R2 (2 μg/mL, 100 μL/well) and mAb M7 (16 μg/mL, 100 μL/well) were used as primary antibodies and incubated at 37°C for 1 h 30 min. Thereafter, 0.1% PBST (400 μL/well) was washed four times, and the secondary antibodies rIgG-HRP (pAb R2; 100 μL/well) and mIgG-HRP (mAb M7; 100 μL/well) was diluted 1:5000 in 0.1% PBST and incubated at 37°C for 1 h. After washing four times with 0.1% PBST (400 μL/well), reacting TMB substrate (100 μL/well) at 37°C for 5 min, treatment with STOP solution (100 μL/well) and it was measured in the ELISA reader of 450 nm. GST was used as a negative control, and GST-S3-P was used as a positive control.

Peptide scanning most often involves the chemical synthesis of overlapping peptides covering the antigen sequence targeted by the investigated antibodies (Geysen et al. 1984). Direct ELISA was performed on pAbs R2 and mAb M7 to observe epitope recognition sites in the synthesized 15-mer peptides. As shown in Figure 4, peptide scanning was performed through direct ELISA to identify epitope sites in mAb M7 using synthesized peptides. The synthesized peptides #29–#33 (213–221 aa) showed binding affinity to mAb M7. In the case of pAb R2 (209–234 aa), it was observed that peptides were recognized in #19 (203–217 aa) to #32 (216–230 aa). These results were consistent with those observed with Western blotting (Figure 2(B)). From these results, it was found that the epitope site of the mAb M7 in rpS3 (185–243 aa) is a specific amino acid sequence (PKDEILPTTPISEQKGGKP). Also, it was observed that pAb R2 has multiple epitopes and one of them has site-specific amino acid sequence of SIVEPKDEILPTTPISEQKGGKPEPP.

**Figure 5.**
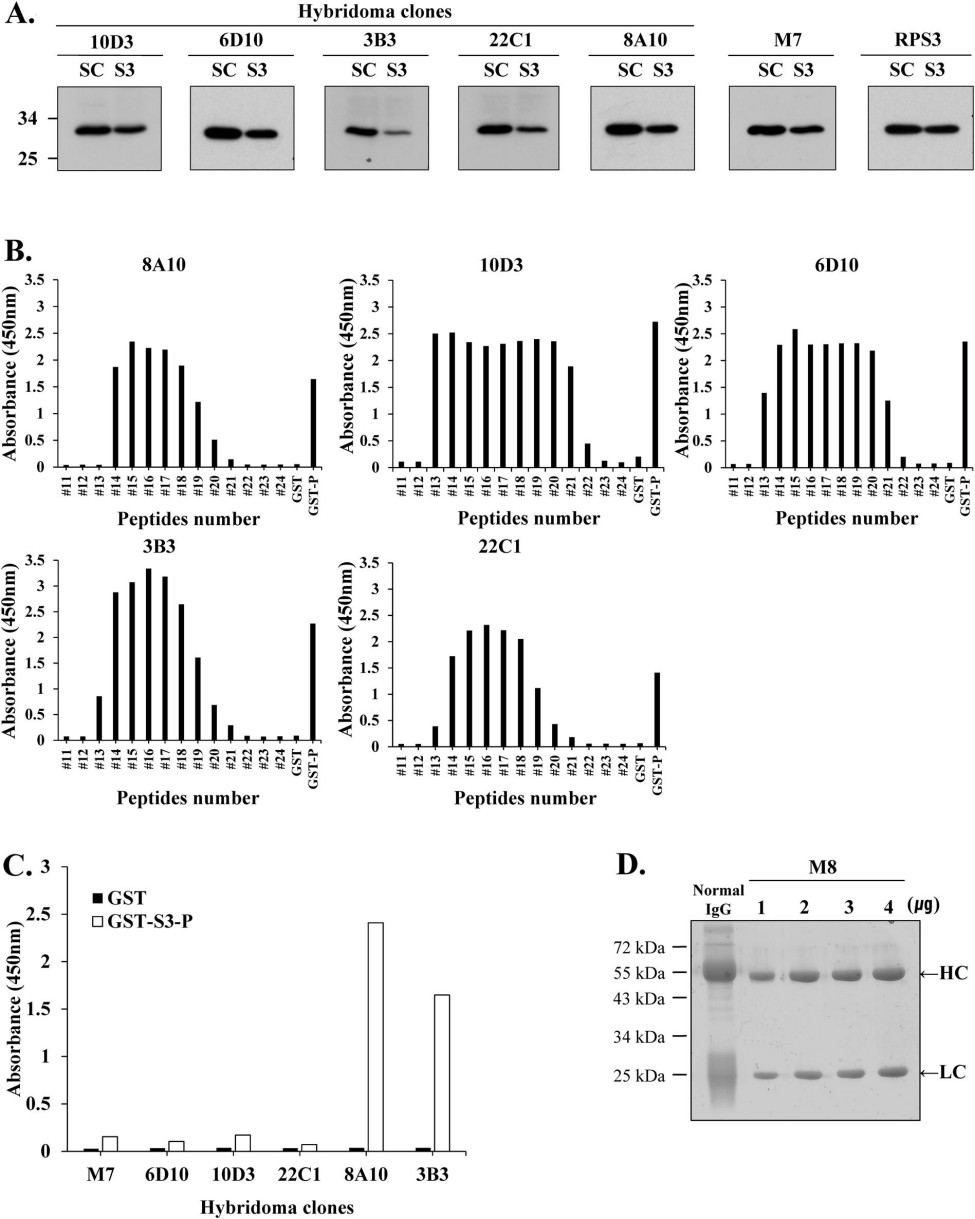
Hybridoma clones for monoclonal antibody production were identified using Western blotting and direct ELISA. (A) For clonal testing of six hybridoma clones, siRNA was transfected into HT1080 cells, and the knockdown of rpS3 was compared with a commercial anti-rpS3 antibody (RPS3) through Western blotting using the hybridoma clone as a primary antibody. mAb M7 was used as a positive control. SC; scramble, control. (B) Epitope identification of hybridoma clones by direct ELISA. (C) Direct ELISA for stable and homogeneous clone selection. GST-S3-P (C-terminal region) was used as a positive control. GST (empty vector) was a negative control. (D) Coomassie blue staining SDS-PAGE analysis for purification of monoclonal antibody M8 against rpS3 after affinity chromatography on Protein A/G Plus column. After purification of mAb M8, samples were analyzed on 12% SDS-PAGE, followed by Coomassie blue staining. Heavy and light chains are indicated by arrows. The positive control was normal IgG.

Hybridomas are engineered to produce monoclonal antibodies by producing large amounts of desired antibodies, and monoclonal antibodies are produced in specialized cells through a technique widely known as hybridoma technology (Zhang 2012; Hanack et al. 2016; Tomita and Tsumoto 2011). Hybridoma clones for monoclonal antibody production were confirmed using Western blotting and direct ELISA. Western blotting was performed to confirm antibody efficacy by comparing each hybridoma clones (10D3, 6D10, 3B3, 22C1, 8A10) and mAb M7 with a commercial anti-rpS3 monoclonal antibody (also named RPS3) (Figure 5(A)). RpS3 exists intracellularly in both mouse and human cancer cells and is indigenously secreted after N-linked glycosylation (Kim et al. 2013; Kim et al. 2016; Park et al. 2019). In this study, HT1080 cells were used for Western blotting and immunoprecipitation assay.

As a result, hybridoma clone M8 confirmed the specific antibody efficacy against rpS3 protein transfected with siRNA in HT1080 cells. In particular, clones 3B3 and 8A10 showed more distinct antibody specificity of rpS3 compared to other clones and mAb RPS3. Direct ELISA revealed that clones 10D3 and 6D10 contained two epitopes and clones 3B3 and 22C1 contained one epitope (Figure 5(B)). For stable and homogeneous clone selection, the supernatant was assayed for reactivity with the corresponding antigen. A single clone with high reactivity to rpS3 was selected through direct ELISA assay for culture and monoclonal antibody purification (Figure 5(C)). As a result of clone selection for antibody purification using direct ELISA assay, 8A10 and 3B3 were found to be suitable. However, 3B3 had better cell conditions than 8A10 and the 3B3 clone was finally selected. Based on these results, mAb M8 produced clone 3B3 through hybridoma technology and was used for future experiments. Culture supernatants of hybridoma 3B3 clone were purified using protein agarose A/G resin, and the following fractions were analyzed by SDS-PAGE with CBB staining (Figure 5(D)).

### MAbs against rpS3 have a higher binding affinity in the GST-S3-P than in His-S3 (FL)

In the previous results, the recognition site of the epitope of mAbs was observed in fragments of the recombinant protein rpS3. The recognition sites of each mAb were identified based on these.

**Figure 6.**
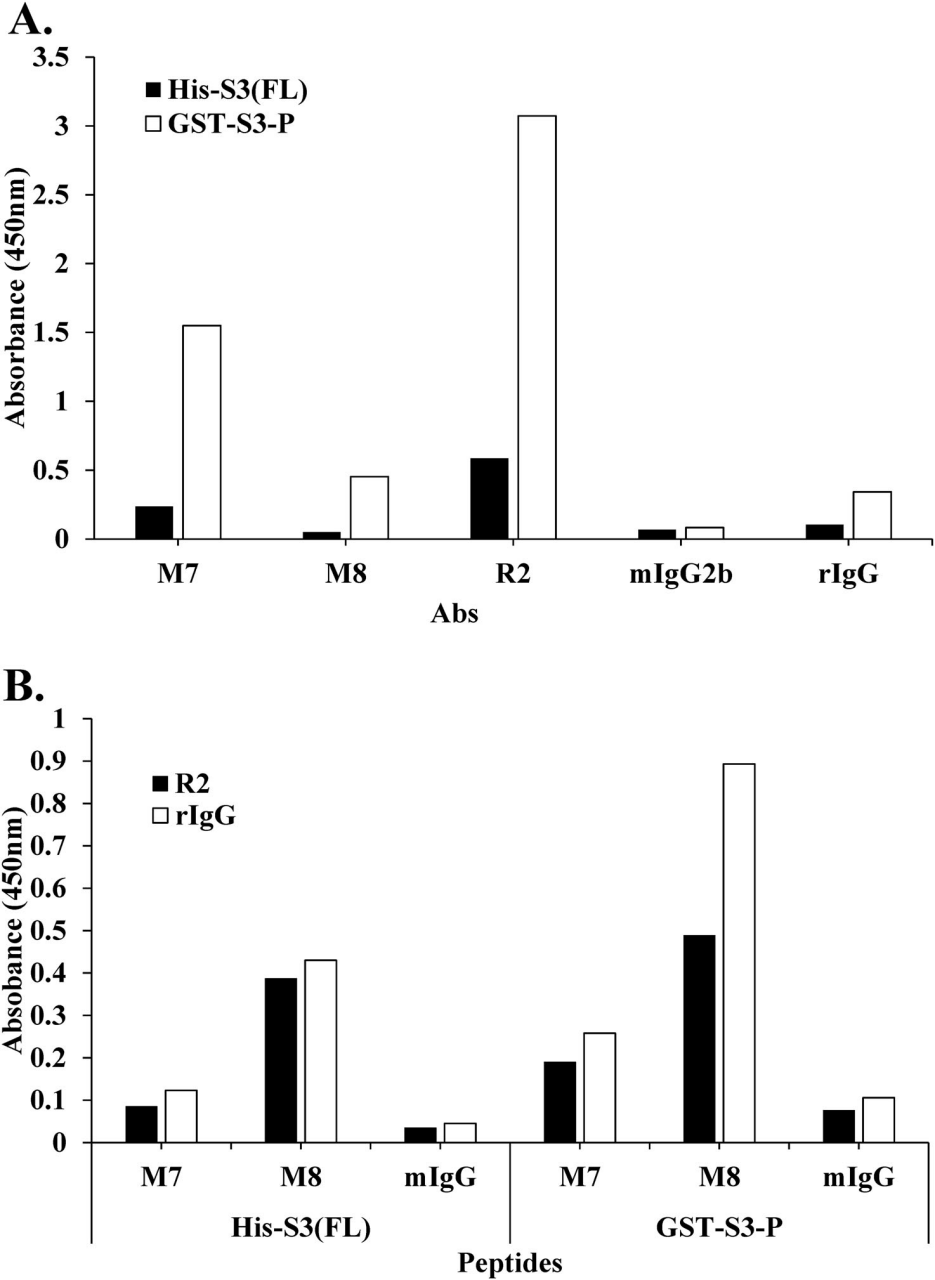
Epitope mapping of mAb M7 and pAb R2 in the recombinant proteins His-S3 (FL) and GST-S3-P (C-terminal region) was performed using direct ELISA and sandwich ELISA. (A) To perform direct ELISA assay, the recombinant protein was coated on a 96-well plate at a concentration of 10 μg/mL (100 μL/well) and incubated at 4°C for overnight. This was blocked with 4% BSA in 0.1% PBST (400 μL/well) at 37°C for 1 h. After washing four times with 0.1% PBST (400 μL/well), 4% BSA in PBST (400 μL/well) was blocked at 37°C for 1 h 30 min. In addition, 0.1% PBST (0.1% Tween 20 in PBS) (400 μL/well) was washed four times and pAbs R2 (2 μg/mL, 100 μL/well), M7, M8, mIgG2b, rIgG (16 μg/mL) as a primary antibody, 100 μL/well) was incubated at 37°C for 1 h 30 min. Thereafter, 0.1% PBST (400 μL/well) was washed four times, and the secondary antibodies rIgG-HRP (pAb R2, rIgG; 100 μL/well) and mIgG-HRP (mAb M7, M8, mIgG2b; 100 μL/well) was diluted 1:5000 in 0.1% PBST and incubated at 37°C for 1 h. After, it was washed three times with PBST. 100 μL/well of TMB substrate was added for color development. Plates were measured at 450 nm by an ELISA plate reader. GST-S3-P was used as a positive control. (B) To perform sandwich ELISA, pAb R2 and rIgG, as capture Abs were diluted to 2 μg/mL in PBS/0.1% Tween 20 (PBST). Then, 100 μL of the solution was coated on the wells of the ELISA plate at 4°C for overnight. Wells were washed four times with 0.1% PBST. 0.1% PBST containing 4% BSA was added at 400 μL/well and incubated at 37°C for 1 h 30 min. Wells were washed four times with PBST. 100 μL of the peptide or recombinant protein diluted at a concentration of 100 ng/mL was added to each well of the plate, and the plate was maintained at 37°C for 1 h. At the end of this period, the plates were washed four times with PBST. The primary antibody, as M7, M8, mIgG (16 μg/mL) was diluted in PBST containing 1% BSA, and 100 μL/well was added. The plate was left at 37°C for 2 h. At the end, the plate was washed three times with PBST. HRP-conjugated rIgG or mIgG was diluted 1:2000 in PBST containing 1% BSA, and 100 μL was added to each well and stored at 37°C for 1 h. After, it was washed three times with PBST. 100 μL/well of TMB substrate was added for color development. Plates were measured at 450 nm by an ELISA plate reader.

As shown in Figure 6, direct ELISA was performed to compare the binding affinity of mAbs in the rpS3 recombinant protein His-S3 (FL) and GST-S3-P (C-terminal region).

Figure 6(A) shows that mAbs M7 and M8, and pAb R2 had higher binding affinity than His-S3 (FL) in GST-S3-P, and mAb M7 showed higher binding affinity than mAb M8 (Figure 6(A)). As a result, these mAbs, such as M7 and M8, were difficult to recognize because the portion to be recognized for each epitope was hidden in a state in which the antigen, rpS3 protein, had a full-length three-dimensional folded structure. That is, the C-terminal region (159-242 aa) of rpS3 was exposed on the surface of the rpS3 protein and could be easily recognized by a mAb. In addition, a sandwich ELISA was performed to confirm competitive Ab binding between pAb R2 and other mAbs. As shown in Figure 6(B), the binding affinity of pAb R2 and two mAbs through sandwich ELISA was higher in GST-S3-P (C-terminal region) than in His-S3 (FL), and in particular, the binding affinities of pAb R2 and two mAbs were higher in GST-S3-P (C-terminal region) than in His-S3 (FL). It was confirmed that mAb M8 showed a higher binding affinity than mAb M7. These results suggested that mAb M8 recognizes the epitope of rpS3 more specifically than mAb M7.

### Test for immunoprecipitation of monoclonal antibodies

**Figure 7.**
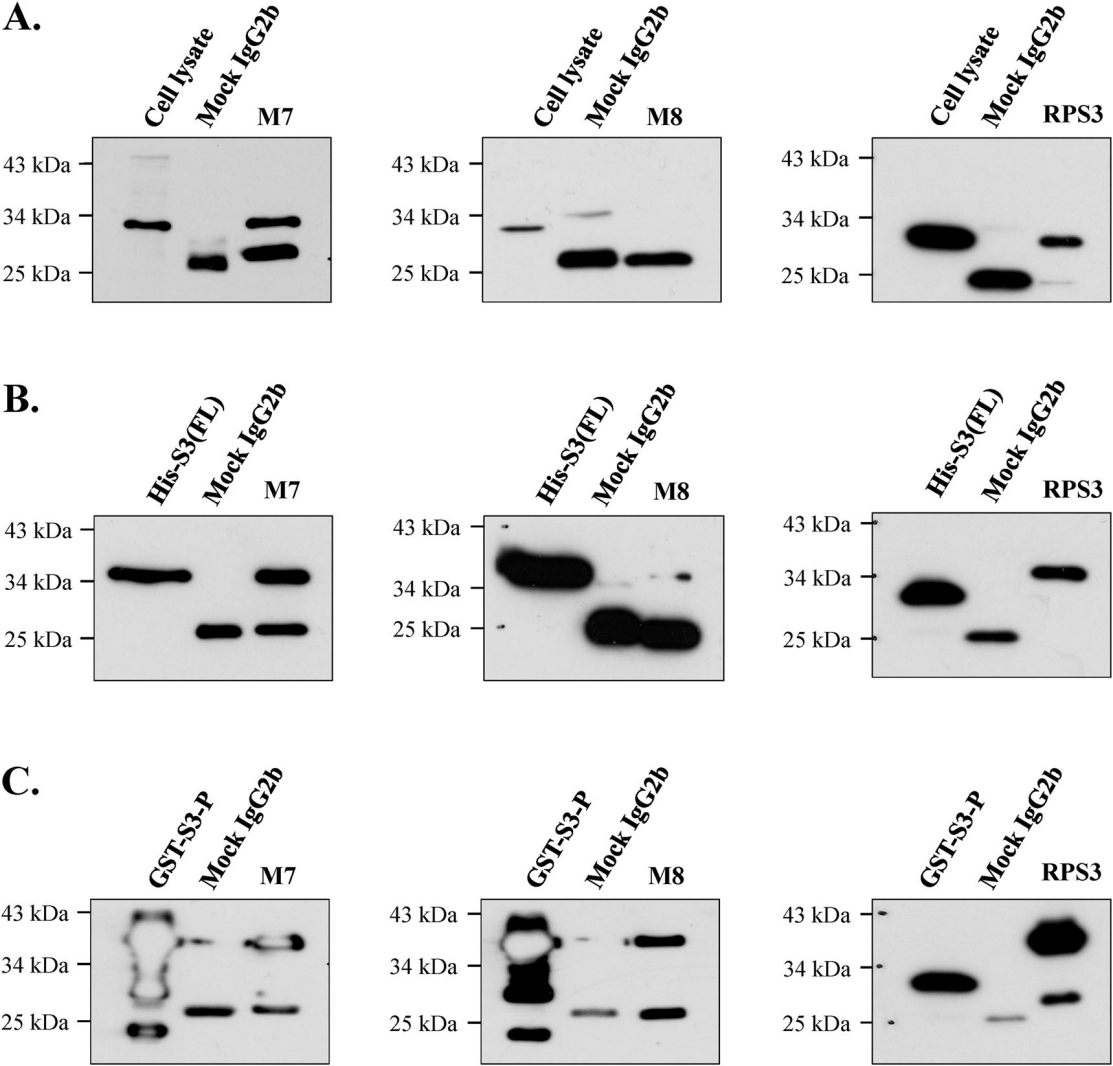
Identification of immunoprecipitation of mAbs in various proteins. The possibility of immunoprecipitation of mAbs M7 and M8 was confirmed through immunoprecipitation assay. (A) To confirm immunoprecipitation, HT1080 cell lysate, (B) and (C) were bacterial cells recombinant protein (His-S3 (FL), GST-S3-P) was performed. The mAbs M7, M8, and mock IgG2b were cross-linked to protein A-Sepharose beads (GE Healthcare) with cell lysis buffer (50 mM Tris-HCl, pH 7.4, 150 mM NaCl, 1% Triton X-100, 0.5% Sodium deoxycholate, 0.1% SDS, 2 mM EDTA). Immunoprecipitated proteins were analyzed by SDS-PAGE and western blotting. Commercial anti-rpS3 monoclonal antibody (RPS3) was used as a positive control.

As shown in Figure 7(A), mAb M7 and commercial anti-rpS3 monoclonal antibody (RPS3) were strongly immunoprecipitated in cell lysates of HT1080 cells, whereas mAb M8 was not immunoprecipitated. On the other hand, in Figure 7(B), it was confirmed that mAb M7 and mAb RPS3 showed strong immunoprecipitation and mAb M8 showed weak immunoprecipitation in recombinant protein His-S3 (FL) expressed in *E. coli.* Also, as shown in Figure 7(C), immunoprecipitation using recombinant bacterial protein GST-S3-P (C-terminal region) showed that mAb M8 was immunoprecipitated at a similar level to that of mAb M7 and mAb RPS3. Based on these results, mAb M7 stably performed immunoprecipitation in most rpS3 protein states, whereas mAb M8 did not (Figure 7(A–C)).

## Discussion

In this study, the recombinant protein rpS3 and a specific epitope in the synthesized peptide were mapped using the produced monoclonal antibodies, and each mAbs and pAb were characterized. We also confirmed that the recombinant protein rpS3 can be applied to immunodetection-based assays, including Western blotting, ELISA, and immunoprecipitation.

Our results revealed a mapping method to identify the epitope of a monoclonal antibody against rpS3. In addition, an epitope-specific Ab was produced using a peptide derived from the C-terminal amino acid of the rpS3 protein, showing its properties and efficacy.

The research and development of monoclonal antibodies is a rapidly progressing field (Aggarwal 2009; Beck et al. 2010). In the past 25 years, more than 30 immunoglobulins (IgGs) and their derivatives have been approved for use in various indications (Beck et al. 2008; Reichert 2010).

MAbs play a variety of roles in the management of cancers, including diagnosis, monitoring, and treatment of diseases. With regards to treatment, these antibodies can enhance the patient’s immune response by acting on specific antigens on cancer cells. MAbs can be programmed to act on cell growth factors to suppress cancer cell growth (Lu et al. 2020; Weiner et al. 2009; Zahavi and Weiner 2020). Alternatively, mAbs may be used in combination with anticancer drugs, radioisotopes, reaction modulators, or toxic substances (Bush 2002). MAbs may also be used to select normal stem cells from the bone marrow or blood in preparation for transplantation of hematopoietic stem cells in cancer patients (Flinn and Lazarus 2001). And, mAbs can be used in applications against cancer cell-specific antigens that will induce an immunological response against the target cancer cell. The availability of mAbs that recognize immune cell antigens has resulted in improved diagnosis of particular types of leukemia and lymphoma. Today, special mAbs are available for colorectal cancer, ovarian cancer, and lung cancers (Beckman et al. 2007; Carter 2001).

Polyclonal antibodies, in contrast, are not as adept as mAbs at treating cancer cells due to their lack of specificity and a high degree of cross-reactivity. Research is showing that pAb therapy can be useful in the treatment of some diseases and as an immunosuppressant for transplant patients (Webster et al. 2006; Webster et al. 2017).

Therapeutic antibodies have made the transition from concept to clinical reality over the past two decades. Many are now being tested as adjuvant or first-line therapies to assess their efficacy in improving or prolonging survival. Modified antibody structures, such as bispecific antibodies or smaller antibody fragments, have yet to claim defined therapeutic roles, whereas radioimmunotherapy has clearly demonstrated efficacy in some disease settings (Maleki et al. 2013).

In conclusion, this study demonstrated that most mAbs and pAb to rpS3 can be mapped by linear, conformational or discontinuous mapping. Furthermore, it can be said that a more in-depth approach is needed to characterize single amino acid properties using micromapping (Sun et al. 2009; Hansen et al. 2013). Through effective epitope generation of these antibodies and the application of in vitro methods, antibodies against rpS3 could serve as biomarkers in cancer diagnosis and treatment.

## Abbreviations

Abbreviations: RPS3, ribosomal protein subunit small 3; mAb, monoclonal antibody; pAb, polyclonal antibody; ELISA, enzyme-linked immunosorbent assay
